# Editorial: Skin microbiome: microbiological, immunological and cellular aspects for therapies to control antimicrobial resistance and skin repair

**DOI:** 10.3389/fcimb.2025.1736430

**Published:** 2025-12-10

**Authors:** Florence Carrouel, Diego Garcia Miranda, Ganeshkumar Arumugam, Marcela Usmari Moraes, Lucas de Paula Ramos

**Affiliations:** 1 Laboratory “Health Systemic Process” (P2S), University Claude Bernard Lyon 1, University of Lyon, Lyon, France; 2 Multimaterials and Interfaces Laboratory, CNRS UMR 5615, University Claude Bernard Lyon 1, University of Lyon, Lyon, France; 3 Department of Biosciences and Oral Diagnosis, Institute of Science and Technology, São Paulo State University, São José dos Campos, Brazil; 4 Department of Materials Physics, Saveetha School of Engineering, Saveetha Institute of Medical and Technical Sciences (SIMTS), Chennai, Tamil Nadu, India; 5 School of Biosciences, College of Health and Life Sciences, Aston University, Birmingham, United Kingdom; 6 School of Dentistry, Federal University of Alfenas-UNIFAL, Alfenas, Brazil

**Keywords:** dermatology, skin microbiome, mycobiome, asepsis, skin abnormalities, skin diseases

The skin, the largest organ of the human body, represents a complex and dynamic ecosystem. Far from being a mere barrier, it hosts a vast and diverse microbiome that communicates continuously with the immune and endocrine systems. This living network supports protection, tolerance, and repair (Byrd et al., 2018). When equilibrium is disturbed, dysbiosis arises, contributing to inflammatory, infectious, or even systemic disorders (De Pessemier et al., 2021). Understanding these microbial and immunological interactions is now central to preventive dermatology and to the broader challenge of controlling antimicrobial resistance (AMR) (O’Neill and Gallo, 2018; Khan and Koh, 2024).

This Research Topic is composed of six complementary studies exploring how skin microbiota, immunity, and innovation converge toward a new paradigm. Their unifying principle is simple: health is not restored by asepsis but by balance.

Czyz et al. revisit the safety and microbiological implications of benzoyl peroxide (BPO) in acne management. Long used for its effectiveness against *Cutibacterium acnes*, BPO has recently been questioned for potential benzene formation. Through careful analysis of toxicological data and regulatory evaluations, the authors conclude that BPO, when appropriately formulated and stored, remains safe and microbiome-compatible. Their paper reframes therapeutic safety as ecological responsibility, showing that antimicrobial efficacy and microbial preservation are not mutually exclusive (Haykal et al., 2024; Scharschmidt and Segre, 2025). By highlighting that commensal equilibrium is crucial for long-term resilience, it also positions AMR prevention within the daily practice of dermatology.

Huang et al. explore the multifaceted interactions between microbial dysbiosis and inflammation in atopic dermatitis. Their comprehensive review details how *Staphylococcus aureus* dominance disrupts the epithelial barrier and triggers Th2-driven inflammation, while *S. epidermidis* and *Malassezia* species contribute to protection and tolerance. The authors emphasize that different therapeutic strategies—from emollients and phototherapy to biologics and microbiota-based products—ultimately converge toward one endpoint: restoration of microbial diversity. This ecological vision aligns with recent efforts to integrate microbiome modulation into the treatment of inflammatory dermatoses.

In the same spirit, Chen et al. demonstrate that dysbiosis and its correction extend beyond inflammatory diseases to viral infections such as *Condyloma acuminatum*. Using 16S rRNA sequencing before and after topical therapy with QYXJ, they observed enrichment of *Streptococcus* and *Caldivirga* and reduced metabolic activity in untreated lesions. Treatment partially normalized microbial composition and restored metabolic pathways involved in lipid and carbohydrate metabolism. This re-symbiosis supports the concept that microbiota modulation can enhance epithelial repair and immune balance, even in viral pathology (Lin et al., 2025; Scharschmidt and Segre, 2025).

Xu et al. provide a methodological contribution with important implications for clinical translation. Comparing three sampling methods—surface swabs, modified biopsies, and comedone extraction—in acne patients, they show that bacterial and fungal profiles vary profoundly with sampling depth. Intrafollicular samples reveal distinct enrichment in *Staphylococcus* and *Malassezia* compared to surface layers. Such findings underscore that the skin microbiome is spatially structured and that reproducible clinical studies require standardized, site-specific methodologies (Pérez-Losada and Crandall, 2023; Smith et al., 2024).

Complementing these mechanistic and methodological perspectives, Mun et al. deliver one of the most integrative analyses to date linking biophysical parameters with microbial ecology. Conducted in nearly one thousand participants, their study combines measurements of hydration, oil content, elasticity, and pigmentation with 16S rRNA sequencing and machine learning. They identify fifteen core bacterial genera—including *Cutibacterium*, *Streptococcus*, *Staphylococcus*, *Rothia*, and *Neisseria*—that define age-related and phenotype-specific “skin cytotypes”. Microbial diversity and composition mirror physical properties of the skin and evolve with aging. Their model predicts skin type with over 90% accuracy, paving the way for precision dermatology and personalized, microbiome-conscious skincare (Sun et al., 2024).

The last contribution, a review on bioactive textiles, broadens the discussion to material science and clinical innovation (Negut et al.). These new fabrics, incorporating bioactive agents such as chitosan, essential oils, plant extracts, or metallic nanoparticles, aim to interact positively with the skin and its microbiota. They selectively inhibit pathogens like *S. aureus* and *C. acnes* while preserving beneficial species such as *S. epidermidis*. By promoting sustainable, biocompatible design and testing within 3D skin models that include commensal flora, the authors propose a new generation of microbiome-safe materials that merge dermatology and biomaterials (de Oliveira and Tavaria, 2024; Ghosh et al., 2025).

Taken together, these six studies articulate a consistent message. Czyz et al. re-examine antimicrobial safety through an ecological lens. Huang et al. place microbial diversity at the heart of immune regulation. Chen et al. demonstrate that restoring microbial metabolism supports epithelial healing. Xu et al. highlight the importance of methodological rigor for reproducible science. Mun et al. integrate physiology and microbial ecology into a predictive framework, and the review on bioactive textiles translates these principles into real-world applications. Collectively, they define the skin microbiome as both a diagnostic and therapeutic partner.

This Research Topic also reframes AMR as an ecological imbalance rather than a pharmacological inevitability. Preserving commensal resilience, restoring microbial function, and supporting metabolic diversity are now key strategies to strengthen the skin’s natural defenses (Ma et al., 2024; Hong et al., 2025). The convergence of microbiology, clinical science, and material innovation opens a path toward sustainable and personalized dermatology.

Ultimately, these studies converge on a single idea: skin health depends on harmony. The future of dermatology will rely not on eradication but on coexistence—on therapies that control pathogens while protecting the microbial allies that maintain barrier integrity. The fight against antimicrobial resistance, the repair of damaged tissues, and the personalization of skincare all rest on the same foundation: restore balance, don’t erase it.
